# Further observations on *Meloidogyne enterolobii* (Nematoda: Meloidogynidae) infecting guava (*Psidium guajava*) in India

**DOI:** 10.21307/jofnem-2020-120

**Published:** 2020-12-14

**Authors:** Tushar Manohar Ghule, Victor Phani, Vishal Singh Somvanshi, Maya Patil, Somnath Bhattacharyya, Matiyar Rahaman Khan

**Affiliations:** 1 Department of Agricultural Entomology, Bidhan Chandra Krishi Viswavidyalaya, Nadia, West Bengal, India; 2 Department of Agricultural Entomology, College of Agriculture, Uttar Banga Krishi Viswavidyalaya, Dakshin Dinajpur, West Bengal, India; 3 Division of Nematology, ICAR – Indian Agricultural Research Institute, New Delhi, India; 4 Department of Genetics and Plant Breeding, Bidhan Chandra Krishi Viswavidyalaya, Nadia, West Bengal, India

**Keywords:** Guava, India, ITS, *Meloidogyne enterolobii*, SCAR

## Abstract

Root-knot nematodes (*Meloidogyne* spp.) infect a large number of crops including guava. We investigated a population of *Meloidogyne* sp. infecting guava in the Coimbatore region of Tamil Nadu, India for identification and species confirmation. Detailed morphological and morphometric observations based on second-stage juveniles, males, females, and perineal patterns showed resemblance of the isolated population with the original and subsequent descriptions of *M. enterolobii*. Isozyme analysis of the young egg-laying females displayed the characteristic esterase phenotype pattern similar to that of *M. enterolobii.* Additionally, the identity of the nematode population was further validated by *M. enterolobii* specific SCAR marker and ITS rDNA. Recently published reports on the occurrence and morphological descriptions of *M. enterolobii* from India are largely incongruent with the original and subsequent redescriptions of the species. Here, we present the most comprehensive morphology and morphometrics of an Indian population of *M. enterolobii* for its authentic identification.

Root-knot nematodes (RKNs, *Meloidogyne* spp.) are global pests comprised of more than 100 nominal species, and attack a large number of host-plants. *Meloidogyne enterolobii* Yang and Eisenback, 1983 is an aggressive RKN species with high reproduction potential that infects a wide range of hosts (Brito et al., 2004). Originally, Yang and Eisenback (1983) described *M. enterolobii* infecting Pacara earpod trees (*Enterolobium contortisiliquum* (Vell.) Morong) from Hainan Island of China. Subsequently, the species has been reported to infect guava (*Psidium guajava* L.) and other important crops causing severe economic losses in many countries (Willers, 1997; Xu et al., 2004; Souza et al., 2006; Kiewnick et al., 2009; Ye et al., 2013; Galbieri et al., 2020). Recognizing the dissemination and damage potential of *M. enterolobii*, especially in the European countries, it has been included in the EPPO alert list (EPPO Bulletin, 2011).

Karssen et al. (2012) revisited the taxonomic status of *M. enterolobii* and concluded it as a senior synonym for *M. mayaguensis,* described by Rammah and Hirschmann (1988) infecting eggplant (*Solanum melongena* L). from Puerto Rico. Morphologically, *M. enterolobii* resembles *M. incognita* (Kofoid and White, 1919) Chitwood, 1949 (Carneiro et al., 2001; Brito et al., 2004). In this context, integrative use of molecular markers, viz., mitochondrial cytochrome c-oxidase subunit I (COI), D2D3 expansion segment of the large subunit 28S rRNA, ribosomal internal transcribed spacer (ITS) region, and the sequence characterized amplified region (SCAR) combined with morphological, morphometric and biochemical (e.g. esterase phenotyping) data provides better resolution for species identification.

We found a severe infestation of *M. enterolobii* in guava in Coimbatore district of Tamil Nadu, India (located at N11°1′6″ and E76°58′21″); and the identity of the species was confirmed by detailed morphology and morphometrics supplemented with biochemical and molecular characterization. In the meantime, several publications that appeared from India reporting the occurrence and redescription of *M. enterolobii* (Poornima et al., 2016; Kumar and Rawat, 2018; Ashokkumar et al., 2019; Suresh et al., 2019) have created uncertain identities of the species. Poornima et al. (2016) reported *M. enterolobii* from Tamil Nadu state causing sudden yellowing and wilting of guava, and identified the species based on morphology, morphometrics and 18S rRNA sequence. But, the report suffers from severe shortcomings, such as the number of specimens used for morphological and morphometric studies was not mentioned, some mean values are out of ranges, and the illustrated perineal pattern was significantly different from the characteristic patterns of *M. enterolobii* (Yang and Eisenback, 1983; Karssen et al., 2012). Ashokkumar et al. (2019) also attempted to characterize *M. enterolobii* infecting guava from nine districts of Tamil Nadu, India, but considerable morphometric disparity was observed with regards to the tail length and gubernaculum length of the males as compared to the original description of *M. enterolobii*. Further, the line drawings depicting the perineal pattern morphology of females; head shape and esophageal region morphology of J2s (seems to be the juveniles of different nematode species); and posterior body morphology of males largely deviate from that of *M. enterolobii sensu stricto*. Suresh et al. (2019) characterized the *M. enterolobii* population infecting guava from three districts of Tamil Nadu, India based on morphological and molecular means; but the photomicrograph and line drawings of the male tail region (seems to be the male of other nematode species), as depicted by the authors, do not match with the original description of *M. enterolobii* (Yang and Eisenback, 1983; Karssen et al., 2012). Additionally, Singh (2020) reported association of *M. enterolobii* to cause serious guava decline problem in the Ratlam district of Madhya Pradesh, India in the presence of *Fusarium oxysporum* f. sp. *psidii*, but the study has not conclusively identified the species. The association of other species of *Meloidogyne* such as *M. incognita, M. javanica* (Treub, 1885) Chitwood, 1949 with guava is not unusual under field conditions. However, the recent reports of RKN problems in guava are unequivocally assigned to *M. enterolobii*. Thus, the available morphological descriptions and illustrations of different *M. enterolobii* population infecting guava in India at this juncture gave rise to serious uncertainties and confusion for its identification.

## Materials and methods

### Morphological characterization

Detailed morphological and morphometric studies of at least 20 or more specimen of females, males and J2s of the RKN species were conducted. The specimens were processed following standard procedures (Seinhorst, 1959; Byrd et al., 1983), and the variations in body shape, anterior end and perineal pattern of females; anterior and posterior end of males and J2s were recorded for characterization purpose (Karssen, 2002; Carneiro et al., 2014; Phani et al., 2018). Measurement data from J2s, males and females were subjected to statistical analyses, and photomicrographs were taken using a Zeiss Axioskop 40 (Carl Zeiss, Oberkochen, Germany) compound light microscope equipped with Canon Power Shot S3 IS camera.

### Biochemical and molecular characterization

Esterase phenotyping assay was performed with 20 egg laying females (dissected out from the infested roots) following standard methodologies (Esbenshade and Triantaphyllou, 1985; Karssen et al., 1995). For molecular characterization, single adult female was lysed using worm lysis buffer (Subbotin et al., 2000), and the ribosomal internal transcribed spacer (ITS 1 and 2) marker was PCR amplified and sequenced as described previously (Vrain et al., 1992; Subbotin et al., 2006). Additionally, the sequence characterized amplified region (SCAR), developed for specific confirmation of *M. enterolobii*, was also amplified and sequenced using the primer pair Me-F (5′-AACTTTTGTGAAAGTGCCGCTG-3′) and Me-R (5′-TCAGTTCAGGCAGGATCAACC-3′) (Long et al., 2006), keeping *M. incognita* and *M. javanica* as positive control (Zijlstra et al., 2000; Meng et al., 2004).

## Results and discussion

The infestation of root-knot nematodes is rapidly expanding in guava growing areas in India; and more than one RKN species have been found to be severely damaging and resulting in decline of orchard with slow-wilting symptom of plants in the presence of other pathogens. A severe infestation of root-knot nematode in guava was reported in the Tamil Nadu state of India (Anonymous, 2016). The distribution of nematode infested planting materials of guava has possibly enabled dissemination and spread of the dreaded nematode species across the country.

**Figure 1:**
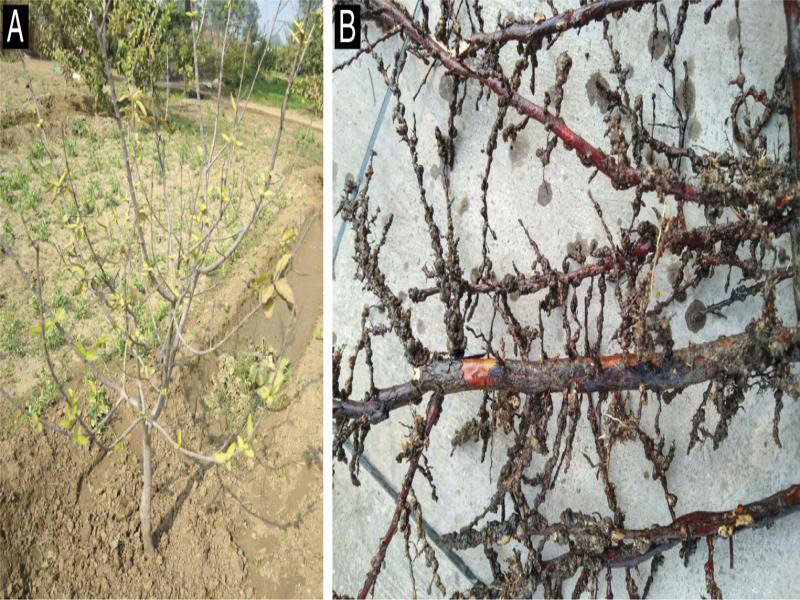
Symptoms induced by *Meloidogyne enterolobii* Yang and Eisenback, 1983 in guava. A: infested guava plant; B: infected plant root showing heavy galling.

**Figure 2:**
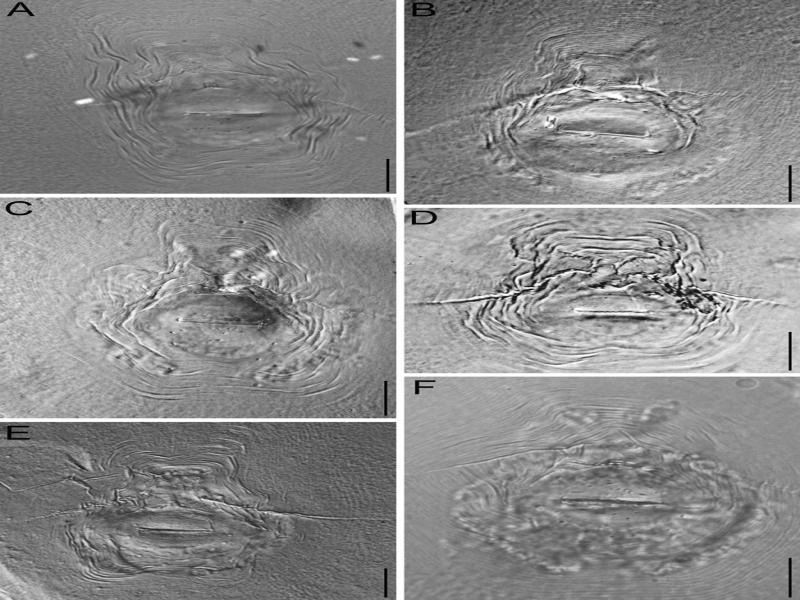
Photomicrographs of perineal patterns of *Meloidogyne enterolobii* Yang and Eisenback, 1983. (scale bar = 20 µm).

**Figure 3:**
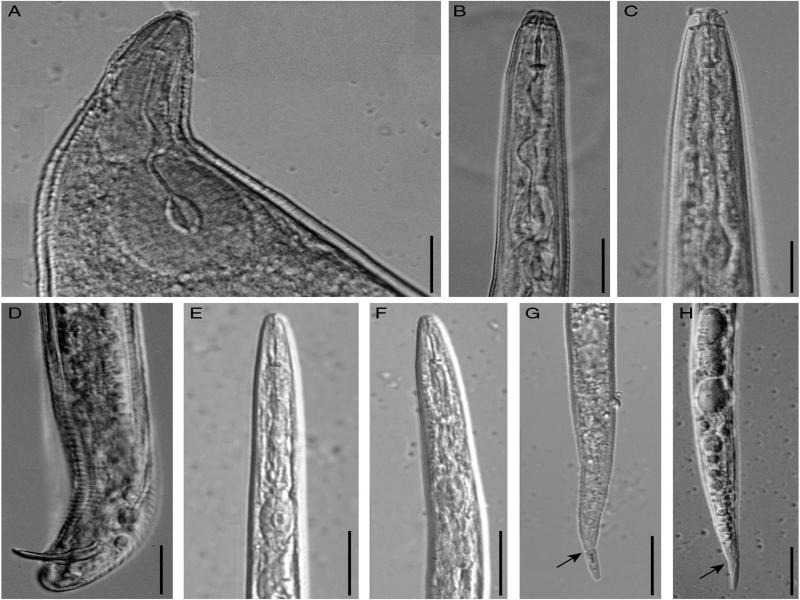
Photomicrographs of female, male, and second-stage juveniles (J2s). A: Anterior end of female; B, C: Anterior ends of male (lateral, dorsal); D: Posterior end of male; E, F: Anterior ends of J2; G, H: Posterior ends of J2 (arrow showing the constriction). (scale bar = 20 µm).

Severe decline of guava with outright crop failure due to the infestation of *M. enterolobii* in commercial guava orchards has been experienced in Brazil (Gomes et al., 2012). Since then, the nematode species is widely known as guava root-knot nematode. The occurrence of *M. enterolobii* in guava has also been reported from other Asian countries like China (Hao et al., 2005) and Vietnam (Iwahori, 2009); but how the nematode species got introduced and established in India still remains elusive. Besides, decline of guava yield and quality has been reported in several parts of Malaysia to be caused by *M. incognita* (Razak and Lim, 1987). Khan et al. (2007) also reported the presence of *M. incognita* and *M. javanica* in the rhizosphere soil of guava in West Bengal state of India, but they were not found to be economically damaging for the crop. While investigating the association of the nematodes in the infested guava plants (Fig. 1A), we found massive deformation (root galling) in the root system (Fig. 1B). Based on the morphometric illustrations of the present population and their simultaneous comparison with earlier descriptions (Yang and Eisenback, 1983; Rammah and Hirschmann, 1988; Karssen et al., 2012) the specific status of *M. enterolobii* was confirmed. We measured the important taxonomic characters of the female anterior end and posterior cuticular patterns and found an overlap of the ranges of all morphometric variables in the Indian population with the reported morphometrics of *M. enetrolobii*. However, mean stylet length (13.4 µm) is slightly shorter than that of previous observations (14.7-15.4 µm). Measurements of vulva-slit (24.8 µm), inter-phasmidal distance (24.6 µm), vulva-anus (20.2 µm) in the perineal pattern are relatively less as compared to the published reports (Table 1). Similarly, in male, distance from the head end to secretor-excretory (SE) pore (130.4 µm) and spicule length (25.2 µm) is relatively less (Table 2). Morphometrics of second-stage juveniles (J2s) are almost similar to the previous descriptions except for the DGO position (2.8 µm), which is slightly closer to the stylet base in the Indian population (Table 3). Morphology of perineal pattern is typical for the species; oval shaped dorsal arch moderately high to high squarish and rounded, with smooth and coarse striae, dorsal and ventral arch forming a weak lateral line, ventral arch broad with smooth striae. However, perineal patterns (Fig. 2A-F) from Indian population of *M. enterolobii* are slightly different (consistently high squarish dorsal arch) from that of Yang and Eisenback (1983), Rammah and Hirschmann (1988), and Karssen et al. (2012). Female (Fig. 3A: anterior end), male (Fig. 3B-D), and J2 (Fig. 3E-H) are also similar for the species. The tail of J2s has a characteristic constriction that could be useful to distinguish it from that of *M. incognita*. The perineal patterns of *M. enterolobii* populations from India are slightly similar to *M. incognita*. Therefore, morphological separation of two closely related species based on the study of few perineal patterns is rather difficult. The difficulty of accurate identification of root-knot nematode species is often compounded by the occurrence of mixture of several species infecting guava. In the present study, we used isozyme and species-specific SCAR markers to confirm the morphological identification.

**Table 1.** Morphometrics of *Meloidogyne enterolobii* Yang and Eisenback, 1983 females from Tamil Nadu, India; compared with earlier descriptions of *M. enterolobii*.

Characters	Present population (n = 20)	*M. enterolobii* Yang and Eisenback, 1983	*M. enterolobii* (Karssen et al., 2012)
Body length	617.0±59.7 (520-710)	735.0±92.8 (541.3-926.3)	693
Body width	465.5±54.8 (380-580)	606.8±120.5 (375.7-809.7)	462
Neck length	–	218.4±74.1 (114.3-466.8)	262
Stylet length	13.4±0.4 (13.1-14.2)	15.1±1.35 (13.2-18.0)	14.7
Stylet knob height	–	2.4±0.26 (1.9-3.1)	2.3
Stylet knob width	–	4.9±0.39 (4.1-5.6)	4.5
Stylet cone	6.3±1.2 (5.6-9.5)	–	–
DEGO	4.3±0.6 (3.7-5.7)	4.9±0.78 (3.7-6.2)	4.8
Length median bulb	36.1±3.3 (31.0-42.7)	–	–
Width median bulb	26.6±4.6 (20.6-35.7)	–	–
Excretory pore to head end	64.9±3.4 (57.7-69.6)	62.9±10.5 (42.3-80.6)	64.0
Length vulva-slit	24.8±1.4 (22.8-27.5)	28.7±2.0 (25.3–32.4)	28.0±1.0 (25.9-29.1)
Inter-phasmid distance	24.6±3.4 (19.0-29.4)	30.7±4.8 (22.2-42.0)	33.5±7.6 (22.4-41.9)
Vulva-anus	20.2±0.9 (19.0-21.8)	22.2±1.8 (19.7-26.6)	23.4±1.6 (21.1-26.2)
Vulval slit-tail terminus	32.9±2.1 (29.4-37.0)	–	–
a	1.3±0.1 (1.1-1.5)	1.2±0.2 (0.9-1.9)	–
Body length/head end to posterior end of metacorpus	–	7.1±0.9 (5.1-9.3)	–

**Note:** All measurements are in μm and in the form: mean ± SD (range).

**Table 2.** Morphometrics of *Meloidogyne enterolobii* Yang and Eisenback, 1983 males from Tamil Nadu, India; compared with earlier descriptions of *M. enterolobii*.

Characters	Present population (*n* = 20)	*M. enterolobii* Yang and Eisenback, 1983	*M. enterolobii* (Karssen et al., 2012)
Body length	1089.1±236.6 (800-1460)	1599.8±159.91 (1348.6-1913.3)	1230±316 (865-1667)
Body width	27.0±3.7 (20.9-33.2)	42.3±3.5 (37.0-48.3)	32.0±6.0 (23.7-39.2)
Tail length	–	12.5±2.24 (8.6-20.2)	11.9±1.2 (10.2-13.4)
Lip height	5.9±0.5 (4.7-6.6)	–	–
Lip width	9.9±0.7 (8.5-11.4)	–	–
Stylet length	21.2±1.3 (19.0-22.8)	23.4±0.96 (21.2-25.5)	21.5±1.7 (19.2-23.4)
Stylet cone	9.7±0.8 (8.5-11.4)	–	–
Stylet shaft	8.3±0.6 (7.1-9.5)	–	–
Stylet knob length	3.2±0.5 (2.3-3.8)	3.3±0.33 (2.6-3.9)	2.5±0.3 (2.1-3.2)
Stylet knob width	4.3±0.4 (3.8-7.6)	5.4±0.34 (4.5-5.8)	4.5±0.6 (3.5-5.0)
DEGO	5.3±1.0 (3.8-7.6)	4.7±0.4 (3.7-5.3)	4.7±0.6 (3.7-5.8)
Head-metacorpus	86.2±7.9 (74.1-101.6)	–	–
Metacarpus length	20.1±2.5 (17.1-25.6)	–	–
Metacarpus width	9.5±0.9 (8.5-11.4)	–	–
Metacarpus-gland end	111.3±12.7 (93.1-133.0)	–	–
Head-SE pore	130.4±21.7 (86.4-153.9)	178.2±11.2 (159.7-206.2)	155.8±22.3 (129.9-199.7)
Spicule length	25.2±4.3 (17.1-31.3)	30.4±1.2 (27.3-32.1)	28.0±1.1 (26.2-29.4)
Gubernaculum length	7.5±0.8 (5.7-8.5)	6.2±1.0 (4.8-8.0)	6.5±0.8 (6.1-8.0)
Testis length	300.9±84.4 (190.0-422.7)	–	–
*a*	40.2±6.0 (32.1-51.2)	37.9± 3.2 (34.1-45.5)	38.1±4.0 (30.0-43.4)
*b″*	12.58±2.21 (9.9-16.1)	–	–
Body length/head end to posterior end of metacorpus	–	15.8±1.32 (13.8-18.4)	-
*c*	–	131.6±24.1 (72.0-173.4)	103.2±23.7 (71.4-135.9)

**Note:** All measurements are in μm and in the form: mean ± SD (range).

**Table 3.** Morphometrics of *Meloidogyne enterolobii* Yang and Eisenback, 1983 second-stage juveniles (J2s) from Tamil Nadu, India; compared with earlier descriptions of *M. enterolobii*.

Character	Present population (*n* = 20)	*M. enterolobii* Yang and Eisenback, 1983	*M. enterolobii* (Karssen et al., 2012)
Body length	413.5±26.7 (375-460)	436.6±16.6 (405.0-472.9)	408±18 (380-442)
Body width	13.9±1.5 (12.3-17.1)	15.3±0.9 (13.9-17.8)	14.8±2.1 (11.0-18.0)
Body width at anus	10.2±1.3 (8.5-13.3)	–	9.8±0.9 (8.0-11.0)
*a*	30.0±3.2 (24.1-35.2)	28.6±1.9 (24.0-32.5)	28.0±3.7 (23.3-34.6)
Stylet length	12.1±0.4 (11.4-12.3)	11.7±0.5 (10.8-13.0)	11.3±0.7 (10.5-13.0)
Stylet knob height	–	1.6±0.1 (1.9-1.8)	1.8±0.3 (1.5-2.0)
Stylet knob width	–	2.9±0.2 (2.4-3.4)	3.0±0.4 (2.5-4.0)
Stylet base to head end	–	–	15.0±0.7 (14.0-16.0)
DGO	2.8±0.3 (2.3-3.8)	3.4±0.3 (2.8-4.3)	3.8±0.3 (3.0-4.5)
Length median bulb	12.3±0.7 (11.4-13.3)	–	–
Width median bulb	7.5±0.9 (6.6-9.5)	–	–
Head-metacorpus	56.1±2.3 (51.3-59.8)	–	–
*b″*	7.3±0.5 (6.3-8.4)	–	–
Head-SE pore	78.9±4.3 (73.1-92.1)	91.7±3.3 (84.0-98.6)	80.8±4.4 (70.0-88.0)
Tail length	50.1±7.0 (42.7-75.1)	56.4±4.5 (41.5-63.4)	52.1±3.4 (45.0-57.0)
*c*	8.3±1.0 (6.1-10.6)	7.8±0.7 (6.8-10.1)	7.9±0.6 (7.0-9.0)
Body length/head end to posterior end of metacorpus	–	6.5±0.1 (6.2-6.9)	–
Hyaline tail terminus length	13.5±1.31 (11.4-16.1)	–	–
*c′*	4.9±0.5 (4.3-5.7)	–	–
Rectum inflated	all	–	–

**Note:** All measurements are in μm and in the form: mean ± SD (range).

**Figure 4:**
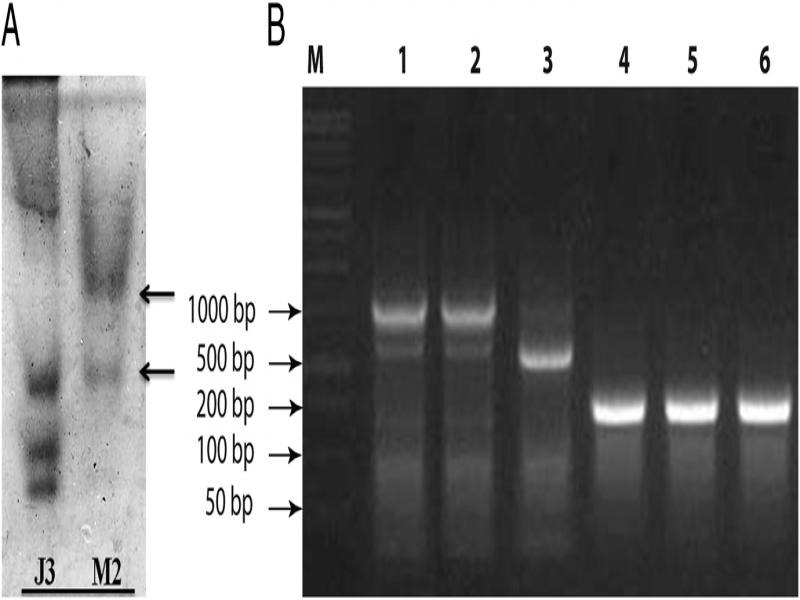
Biochemical and molecular characterization of *Meloidogyne enterolobii* Yang and Eisenback, 1983 population isolated from Tamil Nadu, India. A: Esterase enzyme phenotypic pattern (J3: *M. javanica* (Treub, 1885) Chitwood, 1949; M2: *M. enterolobii* Yang and Eisenback, 1983); B: PCR amplification with SCAR marker (M: DNA weight marker; lanes 1 and 2: *M. incognita* (Kofoid and White, 1919) Chitwood, 1949 control; lane 3: *M. javanica* (Treub, 1885) Chitwood, 1949 control; lanes 4-6: *M. enterolobii* Yang and Eisenback, 1983 populations).

Biochemically, the present RKN population showed species-specific esterase phenotype of *M. enterolobii* (Xu et al., 2004; Paes et al., 2012) (Fig. 4A). Taxonomic identification of *M. enterolobii* has proved to be very challenging based on only the traditional means and has resulted in incorrect identification and misreporting (Brito et al., 2004). Molecular characterization based on ITS rDNA sequences (NCBI GenBank accession number KT271569) and SCAR marker resulted in authentication of the species (Fig. 4B). In this line, use of molecular markers, viz., ribosomal D2D3 expansion segment, ITS rDNA, IGS rDNA, mitochondrial COI, viable region between COII and 16S rDNA, mitochondrial NADH5 and SCAR marker has been found to be more robust for specific confirmation of the species (Tigano et al., 2010; Onkendi and Moleleki, 2013; Rashidifard et al., 2018); and SCAR marker proved best in the situation of occurrence of mixed populations consisting of *M. enterolobii, M. incognita, M. javanica*, and *M. hapla* Chitwood, 1949 (Rashidifard et al., 2018).

In conclusion, here we report detailed morphological and morphometric observations of the Indian *M. enterolobii* population infecting guava. While most of the observations are identical with the earlier descriptions of Yang and Eisenback (1983), Rammah and Hirschmann (1988), and Karssen et al. (2012), some minor variations were observed that could be attributed to differences in hosts, geographical isolates, methodology of specimen processing, artefacts or any microscopic observational bias. The description and illustration in the name of *M. enterolobii* previously reported from India gave rise to the confusion regarding its identification. This study is of cardinal importance for resolving the contention that all RKN infections in guava are *M. enterolobii*. This information would be useful for phenotypic comparisons of various *M. enterolobii* populations from across the world, and might help with the population genetics as well as comparative virulence studies.
